# Longitudinal metabolomic profiling unveils dynamic biomarkers for predicting immune recovery in HIV‐1 infection

**DOI:** 10.1002/ctm2.70502

**Published:** 2025-10-15

**Authors:** Jiali Lv, Fengting Yu, Huiyu Wang, Yu Zhang, Yuqi Wang, Liting Yan, Qing Xiao, Qun Li, Cheng Wang, Shiying Li, Ronghua Jin, Tao Zhang, Junnan Li

**Affiliations:** ^1^ Department of Biostatistics School of Public Health Cheeloo College of Medicine Shandong University Jinan Shandong China; ^2^ National Key Laboratory of Intelligent Tracking and Forecasting for Infectious Diseases Beijing Ditan Hospital Capital Medical University Beijing China; ^3^ Beijing Key Laboratory of Viral Infectious Disease Beijing Ditan Hospital Capital Medical University Beijing China; ^4^ Beijing Key Laboratory of Viral Infectious Disease Beijing Institute of Infectious Disease Beijing China; ^5^ School of Pharmacy Qiqihar Medical University Qiqihar Heilongjiang China; ^6^ Department of Epidemiology and Biostatistics School of Public Health Tianjin Medical University Tianjin China

1

Dear Editor,

Human immunodeficiency virus (HIV) presents a significant public health burden. By 2023, the prevalence of HIV was estimated at 39.9 million globally, while newly acquired infections reached 1.3 million.^1^ Although the widespread use of antiretroviral therapy (ART) has led to a substantial reduction in fatalities linked to HIV/AIDS, approximately 10% to 40% of people living with HIV (PLHIV) still cannot achieve full immune reconstitution despite long‐term viral suppression through ART, and thus face a higher risk of opportunistic infections and mortality.^2^ Metabolic transformation following ART constitutes a multi‐stage abnormal accumulation process in PLHIV. Previous studies have reported several metabolites that could predict the immune response to ART. Nevertheless, prior investigations overlooked metabolite alterations, and were limited by small sample size.^3^

Using dynamic untargeted metabolomics design, fasting plasma was collected pre‐ART and serially at 12, 24, 36, 48, 72, and 96 weeks post‐treatment initiation (*n* = 116) to capture the metabolic transformation following ART, defined as the temporal changes in metabolite profiles (Table 1 and Figure 1A). PLHIV in IR group demonstrated a clearly distinguishable metabolic profile compared to those in the INR group (Figure 1B). Figure 1C revealed 10 significantly changed metabolites after ART, with 8 increased metabolites and 2 decreased metabolites (*p*‐value < .05) (Table S1). The increased metabolites were mostly amino acids, peptides, and analogues, while urate and pantothenate demonstrated monotonic downtrend after ART. According to fuzzy c‐means clustering, two distinct change pattern was found among plasma metabolites, including gradual‐increasing cluster (Cluster 1) and W‐shape cluster (Cluster 2) (Figure 1D).

**TABLE 1:** The clinical characteristics of included PLHIVs by ART immune response.

Variable	IR (*n* = 68)	INR (*n* = 48)	Total (*n* = 116)	*p*‐value
Male, *n* (%)	66 (97.1)	46 (95.8)	112 (96.6)	1.000
Age, years	30 [27, 34]	31 [26.8, 36]	30 [27, 34]	.410
CD4, cells/µL	477.0 [372.5, 538.5]	284.5 [224.0, 361.5]	386.0 [302.5, 512.0]	<.001
VL, copies/mL	27945.5 [8978, 64 513]	28 437 [10 502, 87 569]	27945.5 [9647.2, 71312.2]	.272
WBC, ×10^9^/L	6.4 [5.3, 7.5]	5.6 [4.5, 6.5]	5.9 [4.9, 6.9]	.028
RBC, ×10^12^/L	5.1 [4.8, 5.3]	5.0 [4.8, 5.2]	5.1 [4.8, 5.3]	.640
Hb, g/L	152.0 [143.5, 160.0]	151.0 [141.2, 156.8]	152.0 [142.0, 158.0]	.288
PLT, ×10^9^/L	219.0 [186.5, 250.5]	216.0 [190.2, 244.5]	218.4 [190.0, 246.0]	.446
Cre, µmol/L	67.5 [59.9, 76.0]	67.3 [61.0, 73.2]	67.3 [60.6, 74.1]	.959
ALT, U/L	17.3 [13.2, 25.5]	16.8 [12.4, 23.7]	16.9 [13.0, 25.4]	.627
AST, U/L	19.5 [16.6, 24.6]	20.8 [16.8, 23.5]	20.1 [16.6, 23.9]	.873
TBil, µmol/L	12.6 [9.6, 16.6]	9.9 [8.4, 12.2]	10.8 [8.8, 15.0]	.014
DBil, µmol/L	4.4 [3.2, 6.2]	3.6 [3.1, 4.5]	3.8 [3.1, 5.4]	.021
ALB, g/L	46.9 [45.8, 48.8]	47.1 [45.4, 48.5]	47.0 [45.8, 48.5]	.672
TCho, mmol/L	4.0 [3.5, 4.5]	3.7 [3.5, 4.1]	3.9 [3.5, 4.4]	.106
TG, mmol/L	1.1 [.8, 1.5]	.8 [.7, 1.2]	1.0 [.7, 1.5]	.162
HDL, mmol/L	1.0 [.8, 1.1]	1.0 [.8, 1.1]	1.0 [.8, 1.1]	.503
LDL, mmol/L	2.5 [2.0, 2.9]	2.3 [2.0, 2.6]	2.4 [2.0, 2.8]	.122
ApoA1, g/L	1.2 [1.0, 1.3]	1.1 [1.1, 1.3]	1.2 [1.0, 1.3]	.808
ApoB, g/L	.8 [.7, .9]	.7 [.6, .8]	.8 [.7, .9]	.176
LpA, µmol/L	6.7 [2.7, 20.4]	5.0 [2.9, 11.3]	6.4 [2.8, 14.8]	.986

*Note*: Data are median with interquartile range, or *n* (%).

ALT, alanine aminotransferase; ALB, Albumin; ApoA1, Apolipoprotein A1; ApoB, Apolipoprotein B; ART, antiretroviral therapy; AST, aspartate aminotransferase; CD4, cluster of differentiation 4; Cre, creatinine; D‐BIL, direct bilirubin; Hb, haemoglobin; HDL, high‐density lipoprotein; INR, immunological nonresponse; IR, immunological response; LDL, low‐density lipoprotein; LpA, Lipoprotein A; PLHIVs, people living with HIV; PLT, platelet; RBC, red blood cell; T‐BIL, total bilirubin; TCho, total cholesterol; TG, triglyceride; VL, viral load; WBC, white blood cell.

**FIGURE 1:**
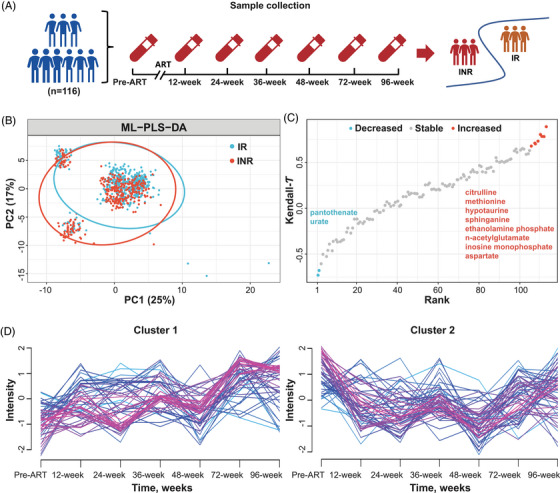
Dynamic metabolomics analysis revealed metabolic transformation after antiretroviral therapy (ART). (A) Overview of the clinical design of the metabolomics study. (B) Multilevel PLS‐DA (ML‐PLS‐DA) score plot discriminating immune inadequate responder (INR) from immune responder (IR) in people living with HIV. (C) Significantly decreased and increased metabolites after ART. Significantly decreased or increased metabolites were selected as the *p*‐value of Mann–Kendall trend test < .05. (D) Two temporal clusters of plasma metabolites, including gradual‐increasing cluster (Cluster 1) and W‐shape cluster (Cluster 2).

In total, five metabolites with the VIP of multilevel PLS‐DA > 1 and *p*‐value tested by linear mixed model < .05 were selected as dynamic differential metabolites (Table S2 and Figure S1). It is worth noting that the plasma arginine levels of PLHIV in the IR group and the INR group not only differed at multiple time points before and after ART, but also exhibited significant differences in dynamic changes after ART (Figure 2A). These findings indicating that focusing solely on the single time point differences in metabolites, while neglecting their dynamic trends, may not fully capture their relationship with the immune response to ART in PLHIV. We further investigated the alteration of clinical characteristics (blood cell, liver function and blood lipids) within 96 weeks after initiating ART, categorised by the immune response of ART (Figure S2). The trajectories of CD4 among PLHIV in both the IR and INR groups exhibit a degree of similarity during the initial 12 to 24 weeks post‐ART. However, these patterns start to diverge after the 36‐week time point. Following the initiation of ART in most PLHIV, blood cell‐related markers exhibited varying degrees of an upward trend.

**FIGURE 2:**
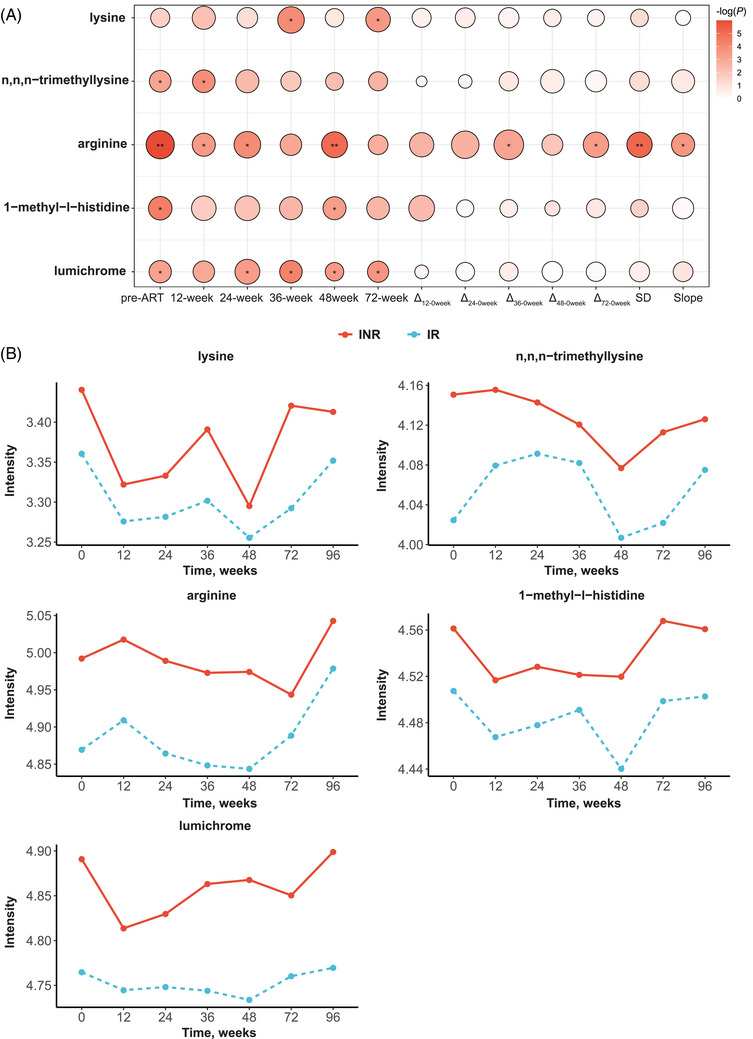
Dynamic differential metabolites discriminating immune inadequate responder (INR). (A) Dynamic differential metabolites to discriminate INR from immune responder (IR) in people living with HIV. Circle colour indicates the –log10(*p*) of Wilcoxon signed rank test, with darker colours representing smaller *p*‐values; Circle size represents the variable importance in projection of PLS‐DA, with larger shapes indicating higher VIP values; *Wilcoxon signed rank test *p*‐value < .05, **Wilcoxon signed rank test *p*‐value < .01. (B) ROC analysis of prediction models to discriminate immune inadequate responder (INR). (C) The AUCs of the delta values for dynamic differential metabolomic biomarkers across different time intervals. (D) The correlations between metabolites‐predicted CD4 count and true CD4 count. *R* ^2^ = *R*‐squared of linear regression model. Note: As 96 weeks is the target time point for CD4 count prediction, we excluded it during metabolite screening to avoid potential bias from overlapping target and predictive time points, so only the 0‐ to 72‐week trend is shown.

Incorporating metabolite change information along with CD4 at different stages of ART treatment effectively enhances the prediction of long‐term immune response (Table S3 and Figure 2B and C). Remarkably, the optimal predictive performance was achieved with metabolite change information at 36 weeks after ART (AUC: .90, 95% CI .83–.95), suggesting that plasma metabolites could serve as a potent tool for early prediction of long‐term immune response to ART. We also explored the ability of dynamic differential metabolites to predict the specific value of CD4 at 96 weeks after ART. In line with this, the metabolic model‐predicted CD4 exhibited a high correlation with the true CD4 (*R* ^2^ = .73, *p*‐value < .001) (Figure 2D).

Metabolic pathway enrichment analysis revealed seven pathways significantly associated with the immune response to ART, including alanine, aspartate and glutamate metabolism, arginine biosynthesis, arginine and proline metabolism, beta‐Alanine metabolism, valine, leucine and isoleucine biosynthesis, glyoxylate and dicarboxylate metabolism, pantothenate and CoA biosynthesis (Figure 3A and Table S4). Among the metabolic pathways associated with the immune response to ART (Figure 3B), glyoxylate and dicarboxylate metabolism, valine, leucine and isoleucine biosynthesis, pantothenate and CoA biosynthesis exhibited decreased activity following ART initiation, then gradually returned to baseline levels at 96 weeks after ART. In contrast, the remaining four pathways showed an overall increasing trend in activity following ART initiation, reaching peak levels at 96 weeks after ART. These findings highlight the dynamic nature of metabolic adaptations during ART and their potential relevance to immune recovery.

**FIGURE 3:**
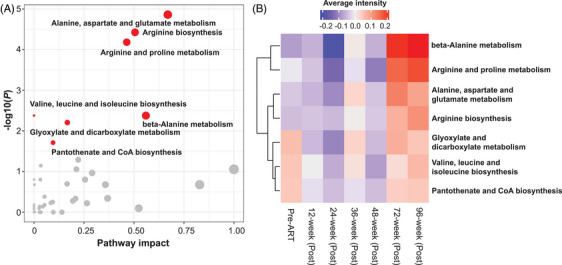
The changes of enriched metabolic pathways in PLHIVs following ART. (A) Metabolic pathways associated with immune response to ART in PLHIVs. Circle colour indicates the –log10(*FDR q value*) of pathway analysis. Red dots denote metabolic pathway associated with ART‐induced immune responses with *FDR q* value < .05. (B) Heatmap shows the temporal changes of metabolic pathways associated with immune response to ART. To quantify pathway activity, the average intensity of metabolites in each pathway at each time window was calculated.

This current study identified arginine as a key metabolite distinguishing IR and INR groups in PLHIV, with arginine pathways emerging as ART‐associated metabolic pathways. Previous study clarified alterations in arginine pathway in PLHIV on ART with virologic suppression, showing independent associations with markers of inflammation and monocyte activation.^4^ Arginine metabolism may contribute to persistent immune activation and inflammation in PLHIV.^5^, ^6^, ^7^ Arginine‐related metabolites alteration also constitutes a modifiable cardiovascular risk factor in in PLHIV,^8^ suggesting that arginine metabolism could serve as a potential mechanism for predicting cardiovascular risk following ART. Future ex vivo studies directly measuring arginine uptake and metabolism in patient‐derived CD4 T cells are warranted to validate this mechanism and explore its therapeutic potential.

Our findings highlight the limitations of single‐time point assessments in evaluating the immune response to ART, whereas tracking metabolite dynamics provides valuable clinical insights for improving ART prognosis in PLHIV. Incorporating longitudinal metabolic changes alongside pre‐ART CD4 count significantly improved the prediction of long‐term immune response, increasing the AUC by 21%. Notably, the optimal predictive performance was achieved with metabolite change information at 36 weeks after ART, suggesting plasma metabolites enable early prediction of long‐term ART immune responses. Although our untargeted metabolomics approach with Level‐1 identification confirmed several significant metabolites with high confidence, a recognised limitation remains the constrained scope of detection. Consequently, while our findings are highly reliable for the metabolites reported, they illuminate only a portion of the complete blood metabolome altered by HIV and ART, and other relevant metabolic pathways may have been undetected. Additionally, without dietary or microbiome data, we are unable to determine the relative contributions of diet, host metabolism, and microbial co‐metabolism to the observed metabolic alterations.

## AUTHOR CONTRIBUTIONS

Study design, JNL, RHJ and TZ; clinical sample collection, FTY, JNL, RHJ; experimental implementation, FTY; data analysis, JNL, JLL, RHJ and TZ; visualisation, JLL, TZ; writing – original draft, JNL, JLL; the corresponding authors, JNL, RHJ, and TZ, had full access to all the data and had final responsibility for the decision to submit it for publication. All authors had full access to all the data in the study and have read and approved the final version of the manuscript.

## CONFLICT OF INTEREST STATEMENT

All authors declare no conflicts of interest with this study.

## FUNDING INFORMATION

This study was supported by grants from R&D Program of Beijing Municipal Education Commission (KM202310025002), National Natural Science Foundation of China (81803288, 82222064 and 82473730) and Heilongjiang Provincial Natural Science Foundation of China (LH2020H132). The funders had no role in study design, data collection and analysis, decision to publish, or preparation of the manuscript.

## ETHICS STATEMENT

This study was approved by the Ethics Committee of the Beijing Ditan Hospital, Capital Medical University. This trial is registered with Center for Drug Evaluation (http://www.chinadrugtrials.org.cn/), number CTR20181797.

## Supporting information



Supporting Information

## Data Availability

All the deidentified participant data for this study, including the individual participant data and a data dictionary for each variable in the study, will be shared upon request for the corresponding author Dr. Junnan Li.

## References

[1] The Joint United Nations Programme on HIV/AIDS. Global HIV & AIDS statistics – Fact sheet. 2023. National Key Laboratory of Intelligent Tracking and Forecasting for Infectious Diseases, Beijing Ditan Hospital, Capital Medical University, Beijing 100015, China. doi:https://www.unaids.org/en/resources/fact‐sheet

[2] Zhang F , Dou Z , Ma Y , et al. Effect of earlier initiation of antiretroviral treatment and increased treatment coverage on HIV‐related mortality in China: a national observational cohort study. Lancet Infect Dis. 2011;11(7):516‐524. doi:10.1016/S1473-3099(11)70097-4 21600849

[3] Pasternak AO , Grijsen ML , Wit FW , et al. Cell‐associated HIV‐1 RNA predicts viral rebound and disease progression after discontinuation of temporary early ART. JCI Insight. 2020;5(6). doi:10.1172/jci.insight.134196 PMC7213790 32097124

[4] Dirajlal‐Fargo S , Alam K , Sattar A , et al. Comprehensive assessment of the arginine pathway and its relationship to inflammation in HIV. AIDS. 2017;31(4):533‐537. doi:10.1097/QAD.0000000000001363 27922857 PMC5263146

[5] Kurz K , Teerlink T , Sarcletti M , Weiss G , Zangerle R , Fuchs D . Plasma concentrations of the cardiovascular risk factor asymmetric dimethylarginine (ADMA) are increased in patients with HIV‐1 infection and correlate with immune activation markers. Pharmacol Res. 2009;60(6):508‐514. doi:10.1016/j.phrs.2009.07.009 19651212

[6] Hudson CL , Zemlin AE , Ipp H . The cardiovascular risk marker asymmetric dimethylarginine is elevated in asymptomatic, untreated HIV‐1 infection and correlates with markers of immune activation and disease progression. Clin Biochem. 2014;51:568‐575. doi:10.1177/0004563213505848 24142400

[7] Hattab S , Guiguet M , Carcelain G , et al. Soluble biomarkers of immune activation and inflammation in HIV infection: impact of 2 years of effective first‐line combination antiretroviral therapy. HIV Med. 2015;16(9):553‐562. doi:10.1111/hiv.12257 25944318

[8] Tang WH , Wang Z , Cho L , Brennan DM , Hazen SL . Diminished global arginine bioavailability and increased arginine catabolism as metabolic profile of increased cardiovascular risk. J Am Coll Cardiol. 2009;53(22):2061‐2067. doi:10.1016/j.jacc.2009.02.036 19477356 PMC2755213

