# Supplementary material for: Longitudinal metabolomic profiling unveils dynamic biomarkers for predicting immune recovery in HIV‐1 infection
Source: Clin Transl Med. 2025 Oct 15;15(10):e70502. doi: 10.1002/ctm2.70502 (PMC12521791; doi:10.1002/ctm2.70502)
Supplement: Supplementary file 1 — Supporting Information [file CTM2-15-e70502-s001.docx]

**Supplemental Materials**

**Supplemental Methods**

**Supplemental Table 1.** Analysis of Monotonic Trends in Plasma Metabolites

**Supplemental Table 2.** The selection of differential metabolites for immune response to ART

**Supplemental Table 3.** ROC analysis of random forest model to predict ART immune response

**Supplemental Table 4.** Result from pathway analysis

**Supplemental Figure 1.** Different temporal patterns of dynamic differential metabolites and clinical characteristics between IR and INR

**Methods**

**Study cohort**

Based on dynamic metabolomics study design, a total of 116 individuals newly diagnosed with HIV infection were enrolled in this study from Beijing Ditan Hospital, Capital Medical University. The following inclusion criteria were considered: (1) newly diagnosed with HIV infection; (2) older than 18 years old; (3) scheduled to receive antiretroviral therapy; (4) above 1000 HIV RNA copies/ml prior to enrollment, and all of which had to be below 40 HIV RNA copies/ml or undetected at 96 weeks after ART; (5) informed consents were obtained before inclusion. Exclusion criteria included (1) taking corticosteroids drugs; (2) diagnosed with acute HIV infection; (3) suffering from AIDS related diseases; (4) co-infection of tuberculosis.

All enrolled participants were treated with a combination of two nucleoside reverse transcriptase inhibitors (NRTI) plus a non-NRTI (NNRTI) and/or a protease inhibitor or a combination of both during follow-up duration. PLHIV whose CD4+ T cells >500 cells/μL at 96 weeks after ART were diagnosed as complete immune responders (IRs), or else were viewed as inadequately respond to ART (INRs)^1^. This study was approved by the Ethics Committee of the Beijing Ditan Hospital, Capital Medical University. This trial is registered with Center for Drug Evaluation (http://www.chinadrugtrials.org.cn/), number CTR20181797.

**Collection of plasma samples**

Plasma samples of the enrolled participants were collected before ART (n=116), 12-week after ART (n=116), 24-week after ART (n=116), 36-week after ART (n=116), 48-week after ART (n=115), 72-week after ART (n=110) and 96-week after ART (n=106). Finally, 795 fasting plasma samples from 116 PLHIV were collected during follow-up duration. All the plasma samples were thawed at 4°C, followed by vortexing for 30 seconds. Quality control (QC) samples were obtained by mixing all enrolled samples by equal amounts. For extraction of metabolites, to 50 µL of serum, 150 µL of methanol (containing internal standards) was added, followed by vortexing for 1 min. Then, 500 µL of MTBE was added and vortexed for another minute. Next, 125 µL of deionized water was added to the mixture, which was then shaken for 10 minutes. The mixture was centrifuged at 12,000 rpm for 10 minutes, and 200 µL of the lower-phase supernatant was collected and dried.

**LC-MS-based metabolomics**

The chromatographic separation using a UHPLC system (Waters, Milford, MA, USA) equipped with a column of HSS T3 2.1 mm × 100 mm, 1.8m (Waters, Milford, MA). The mobile phase B was deionized water with 0.1% formic acid while mobile phase A consisted of acetonitrile containing 0.1% formic acid. Prior to analysis, the residue was dissolved with 100uL of 20/80 (v/v) acetonitrile-water solution. After being centrifuged at 14000 g, 4 °C for 15 min, the supernatant was isolated and 3μL was injected. The flow rate was 0.3 mL/min and the gradient for separation was compiled as follows: 1-70% A from 0.01 to 6 min; 70-100% A from 6 to 6.5 min, followed by maintaining 100% A for 2 min; changing the A back to 1% from 8.5 to 9 min, followed by an equilibration for 2 min. During the analyses, the column temperature was kept at 40 °C.

Data acquisition was carried out by using a 6520 series quadruple time-of-flight mass spectrometer (Agilent, Santa Clara, USA). The conditions for data acquisition were set as follows: spray voltage: 4kV for position ion mode and 3.5 kV for negative ion mode; nebulization gas temperature and flow rate: 330°C, 10 L/min; skimmer and fragmentor voltages: 100 V and 75 V; scan range: m/z 70~1100; scan rate: 3 spectra/second. TripleTOF 4600 series quadruple time-of-flight mass spectrometer (AB Sciex, Redwood City, CA) in both positive and negative ion modes. The parameters for acquisition were set as follows: Spray voltages: 5.5 kV for positive ion mode while 5.0 kV for negative ion mode; TEM was set as 550 °C and both GS1 and GS2 were 55 psi. The acquisition was conducted in full scan mode with a scanning rate of 3 spectra/s and mass range ranged from m/z 50 to 1000.

To ensure data stability, isotopic metabolites were used as internal standards for data normalization. Additionally, quality control runs were inserted after every 10 analytical samples in each batch to monitor data stability. The variation of each metabolite across QC samples was evaluated, and only metabolites with a variation coefficient of less than 30% were retained for further analysis.

**Metabolite identification and preprocessing**

A total of 123 metabolites with level 1 identification were used for subsequent analyses. According to the definition of metabolomics standards initiative (MSI), level 1 means metabolites annotated through matching of accurate precursor m/z (MS1), retention time (RT) and tandem MS/MS spectra (MS2) with the standard metabolite library, with retention time tolerance set to 0.2 minutes and m/z errors within 5 ppm. All these identified metabolites were validated using authentic standards. For each metabolite, K-nearest neighbor (KNN) algorithm was applied to missing values imputation. Metabolites with a missing rate of ≥20% were excluded from the analysis (n=10). In total, 113 metabolites were included in the subsequent analysis, with all QC samples exhibiting an RSD of less than 30%.

**Statistical methods**

The Mann-Kendall trend test was employed to assess whether plasma metabolites exhibited consistent trends of increase or decrease over time (R package: ‘trend’). A monotonic trend was considered significant if the *P*-value of the Mann-Kendall trend test was <0.05. The strength of trends was determined by the absolute value of the Kendall-*Ƭ* value. Subsequently, temporal change clusters of plasma metabolites after ART were elucidated through fuzzy c-means clustering (R package: ‘Mfuzz’)^2^.

Multilevel partial least-squares discriminant analysis (ML-PLS-DA) and linear mixed effect model were utilized for dynamic differential metabolite selection (R package: ‘mixOmics’)^3^. The linear mixed-effects model was fitted for each metabolite with formula: metabolites ~ 1 | patient ID | response + sex + age + CD4 cell count (pre-ART) + time + response *time. Fixed effects included the immune response to ART, sex, age, CD4 cell count (pre-ART), time-dependent effects, and interactions between immune response group and time, with patient ID as a random effect. The threshold criteria of dynamic differential metabolites was set as the VIP of ML-PLS-DA >1 & the fixed effect term *P*-value of ART immune response <0.05. Changes in dynamic differential metabolites were quantified by calculating the difference between pre- and post-ART measurements (Δ_72-0week_, Δ_48-0week_, Δ_36-0week_, Δ_24-0week_, Δ_12-0week_). The area under the receiver operating characteristic curve (AUC) was computed based on leave-one-out cross validation to evaluate the discrimination performance of random forest model.

To enhance our understanding of the mechanisms underlying ART immune response, we conducted the metabolic pathway analysis based on Kyoto Encyclopedia of Genes and Genomes and the Small Molecule Pathway Database (<https://github.com/xia-lab/MetaboAnalystR>)^4^. The statistical significance of pathways’ activity was evaluated by global testing with the default method.

**Reference**

1. Yang X, Su B, Zhang X, Liu Y, Wu H, Zhang T. Incomplete immune reconstitution in HIV/AIDS patients on antiretroviral therapy: Challenges of immunological non-responders. *J Leukoc Biol*. Apr 2020;107(4):597-612. doi:10.1002/JLB.4MR1019-189R

2. Futschik ME, Carlisle B. Noise-robust soft clustering of gene expression time-course data. *J Bioinform Comput Biol*. Aug 2005;3(4):965-88. doi:10.1142/s0219720005001375

3. Welham Z, Dejean S, Le Cao KA. Multivariate Analysis with the R Package mixOmics. *Methods Mol Biol*. 2023;2426:333-359. doi:10.1007/978-1-0716-1967-4_15

4. Chong J, Soufan O, Li C, et al. MetaboAnalyst 4.0: towards more transparent and integrative metabolomics analysis. *Nucleic Acids Res*. Jul 2 2018;46(W1):W486-W494. doi:10.1093/nar/gky310

**Supplemental Table 1.** Analysis of Monotonic Trends in Plasma Metabolites

| **Metabolites** | ***z*** | ***Ƭ*** | ***P*** | **Rank** | **color** |
| --- | --- | --- | --- | --- | --- |
| **citrulline** | **0.905** | **0.905** | **0.007** | **113** | **Increased** |
| **methionine** | **0.810** | **0.810** | **0.016** | **112** | **Increased** |
| **hypotaurine** | **0.810** | **0.810** | **0.016** | **111** | **Increased** |
| **sphinganine** | **0.810** | **0.810** | **0.016** | **110** | **Increased** |
| **ethanolamine phosphate** | **0.714** | **0.714** | **0.035** | **109** | **Increased** |
| **n-acetylglutamate** | **0.714** | **0.714** | **0.035** | **108** | **Increased** |
| **inosine monophosphate** | **0.714** | **0.714** | **0.035** | **107** | **Increased** |
| **aspartate** | **0.714** | **0.714** | **0.035** | **106** | **Increased** |
| histidine | 0.619 | 0.619 | 0.072 | 105 | Stable |
| nicotinamide | 0.619 | 0.619 | 0.072 | 104 | Stable |
| taurine | 0.619 | 0.619 | 0.072 | 103 | Stable |
| 4-guanidinobutanoate | 0.619 | 0.619 | 0.072 | 102 | Stable |
| deoxycarnitine | 0.619 | 0.619 | 0.072 | 101 | Stable |
| glutamate | 0.619 | 0.619 | 0.072 | 100 | Stable |
| theophylline | 0.619 | 0.619 | 0.072 | 99 | Stable |
| phosphorylcholine | 0.619 | 0.619 | 0.072 | 98 | Stable |
| 1-methyladenosine | 0.619 | 0.619 | 0.072 | 97 | Stable |
| methyl 4-aminobutyrate | 0.524 | 0.524 | 0.133 | 96 | Stable |
| 10-hydroxydecanoate | 0.524 | 0.524 | 0.133 | 95 | Stable |
| 1-aminocyclopropanecarboxylate | 0.524 | 0.524 | 0.133 | 94 | Stable |
| threonine | 0.524 | 0.524 | 0.133 | 93 | Stable |
| oxoproline | 0.524 | 0.524 | 0.133 | 92 | Stable |
| trigonelline | 0.524 | 0.524 | 0.133 | 91 | Stable |
| n-methylglutamate | 0.524 | 0.524 | 0.133 | 90 | Stable |
| glucosamine 6-phosphate | 0.524 | 0.524 | 0.133 | 89 | Stable |
| n-acetylneuraminate | 0.524 | 0.524 | 0.133 | 88 | Stable |
| succinate | 0.429 | 0.429 | 0.230 | 87 | Stable |
| 2-hydroxy-4-(methylthio)butanoate | 0.429 | 0.429 | 0.230 | 86 | Stable |
| trans-4-hydroxy-l-proline | 0.429 | 0.429 | 0.230 | 85 | Stable |
| hypoxanthine | 0.429 | 0.429 | 0.230 | 84 | Stable |
| spermidine | 0.429 | 0.429 | 0.230 | 83 | Stable |
| n,n-dimethyl-arginine | 0.429 | 0.429 | 0.230 | 82 | Stable |
| spermine | 0.429 | 0.429 | 0.230 | 81 | Stable |
| tryptophan | 0.429 | 0.429 | 0.230 | 80 | Stable |
| uridine | 0.429 | 0.429 | 0.230 | 79 | Stable |
| galactose 1-phosphate | 0.429 | 0.429 | 0.230 | 78 | Stable |
| lauroylcarnitine | 0.429 | 0.429 | 0.230 | 77 | Stable |
| bis(2-ethylhexyl)phthalate | 0.429 | 0.429 | 0.230 | 76 | Stable |
| benzoate | 0.333 | 0.333 | 0.368 | 75 | Stable |
| d-alanine | 0.333 | 0.333 | 0.368 | 74 | Stable |
| proline | 0.333 | 0.333 | 0.368 | 73 | Stable |
| l-ornithine | 0.333 | 0.333 | 0.368 | 72 | Stable |
| l-carnitine | 0.333 | 0.333 | 0.368 | 71 | Stable |
| hippurate | 0.333 | 0.333 | 0.368 | 70 | Stable |
| deoxyadenosine | 0.333 | 0.333 | 0.368 | 69 | Stable |
| quinate | 0.238 | 0.238 | 0.548 | 68 | Stable |
| deoxycholate | 0.238 | 0.238 | 0.548 | 67 | Stable |
| succinate semialdehyde | 0.238 | 0.238 | 0.548 | 66 | Stable |
| serine | 0.238 | 0.238 | 0.548 | 65 | Stable |
| diethanolamine | 0.238 | 0.238 | 0.548 | 64 | Stable |
| pipecolate | 0.238 | 0.238 | 0.548 | 63 | Stable |
| isoleucine | 0.238 | 0.238 | 0.548 | 62 | Stable |
| 4-coumarate | 0.238 | 0.238 | 0.548 | 61 | Stable |
| phenylalanine | 0.238 | 0.238 | 0.548 | 60 | Stable |
| indole-3-acetate | 0.238 | 0.238 | 0.548 | 59 | Stable |
| n-alpha-acetyllysine | 0.238 | 0.238 | 0.548 | 58 | Stable |
| indole-3-methyl acetate | 0.238 | 0.238 | 0.548 | 57 | Stable |
| isocitrate | 0.238 | 0.238 | 0.548 | 56 | Stable |
| glutarylcarnitine | 0.238 | 0.238 | 0.548 | 55 | Stable |
| glycochenodeoxycholate | 0.238 | 0.238 | 0.548 | 54 | Stable |
| indole | 0.143 | 0.143 | 0.764 | 53 | Stable |
| glyceraldehyde | 0.143 | 0.143 | 0.764 | 52 | Stable |
| aminoisobutanoate | 0.143 | 0.143 | 0.764 | 51 | Stable |
| creatinine | 0.143 | 0.143 | 0.764 | 50 | Stable |
| betaine | 0.143 | 0.143 | 0.764 | 49 | Stable |
| n-acetylalanine | 0.143 | 0.143 | 0.764 | 48 | Stable |
| lysine | 0.143 | 0.143 | 0.764 | 47 | Stable |
| histidine | 0.143 | 0.143 | 0.764 | 46 | Stable |
| dihydroxyacetone phosphate | 0.143 | 0.143 | 0.764 | 45 | Stable |
| n-acetylleucine | 0.143 | 0.143 | 0.764 | 44 | Stable |
| lumichrome | 0.143 | 0.143 | 0.764 | 43 | Stable |
| galactarate | 0.048 | 0.048 | 1.000 | 42 | Stable |
| valine | 0.048 | 0.048 | 1.000 | 41 | Stable |
| pyroglutamate | 0.048 | 0.048 | 1.000 | 40 | Stable |
| leucine | 0.048 | 0.048 | 1.000 | 39 | Stable |
| asparagine | 0.048 | 0.048 | 1.000 | 38 | Stable |
| xanthine | 0.048 | 0.048 | 1.000 | 37 | Stable |
| arginine | 0.048 | 0.048 | 1.000 | 36 | Stable |
| n-acetylglucosamine | 0.048 | 0.048 | 1.000 | 35 | Stable |
| glycocholate | 0.048 | 0.048 | 1.000 | 34 | Stable |
| malate | -0.048 | -0.048 | 1.000 | 33 | Stable |
| lactate | -0.048 | -0.048 | 1.000 | 32 | Stable |
| 2-aminoisobutyrate | -0.048 | -0.048 | 1.000 | 31 | Stable |
| n-acetylputrescine | -0.048 | -0.048 | 1.000 | 30 | Stable |
| glutamine | -0.048 | -0.048 | 1.000 | 29 | Stable |
| alpha-hydroxyisobutyrate | -0.143 | -0.143 | 0.764 | 28 | Stable |
| glycerate | -0.143 | -0.143 | 0.764 | 27 | Stable |
| allantoin | -0.143 | -0.143 | 0.764 | 26 | Stable |
| suberate | -0.143 | -0.143 | 0.764 | 25 | Stable |
| citrate | -0.143 | -0.143 | 0.764 | 24 | Stable |
| glucuronate | -0.143 | -0.143 | 0.764 | 23 | Stable |
| 1-methyl-l-histidine | -0.143 | -0.143 | 0.764 | 22 | Stable |
| 4-pyridoxate | -0.143 | -0.143 | 0.764 | 21 | Stable |
| l-alanine | -0.143 | -0.143 | 0.764 | 20 | Stable |
| beta-alanine | -0.143 | -0.143 | 0.764 | 19 | Stable |
| 4-hydroxybenzoate | -0.238 | -0.238 | 0.548 | 18 | Stable |
| n,n,n-trimethyllysine | -0.238 | -0.238 | 0.548 | 17 | Stable |
| 3-methoxytyrosine | -0.238 | -0.238 | 0.548 | 16 | Stable |
| cystine | -0.238 | -0.238 | 0.548 | 15 | Stable |
| n-acetylaspartate | -0.333 | -0.333 | 0.368 | 14 | Stable |
| n-formyl-l-methionine | -0.333 | -0.333 | 0.368 | 13 | Stable |
| gluconate | -0.333 | -0.333 | 0.368 | 12 | Stable |
| ureidopropionate | -0.333 | -0.333 | 0.368 | 11 | Stable |
| homocysteine | -0.333 | -0.333 | 0.368 | 10 | Stable |
| n-acetylproline | -0.333 | -0.333 | 0.368 | 9 | Stable |
| tyrosine | -0.429 | -0.429 | 0.230 | 8 | Stable |
| kynurenine | -0.429 | -0.429 | 0.230 | 7 | Stable |
| palmitoylcarnitine | -0.429 | -0.429 | 0.230 | 6 | Stable |
| creatine | -0.524 | -0.524 | 0.133 | 5 | Stable |
| 5-hydroxytryptophan | -0.524 | -0.524 | 0.133 | 4 | Stable |
| cortisol | -0.619 | -0.619 | 0.072 | 3 | Stable |
| **urate** | **-0.714** | **-0.714** | **0.035** | **2** | **Decreased** |
| **pantothenate** | **-0.714** | **-0.714** | **0.035** | **1** | **Decreased** |

*P* = The *P* value of MK test, Ƭ = The Kendall-Ƭ value of MK test, *z* = The *z* value of MK test.

**Supplemental Table 2.** The selection of differential metabolites for immune response

| **Metabolite** | ***P*-value** | **VIP** | **m/z** | **RT** | **Cluster** |
| --- | --- | --- | --- | --- | --- |
| **lumichrome** | **0.010** | **1.52** | **4.40** | **243.09** | **2** |
| **1-methyl-l-histidine** | **0.010** | **1.33** | **0.81** | **170.09** | **2** |
| **arginine** | **0.013** | **1.59** | **0.79** | **175.12** | **1** |
| **n,n,n-trimethyllysine** | **0.044** | **1.20** | **0.80** | **189.16** | **2** |
| **lysine** | **0.046** | **1.26** | **0.71** | **147.11** | **2** |
| 4-pyridoxate | 0.047 | 0.96 | 2.21 | 184.06 | 2 |
| hippurate | 0.054 | 0.79 | 3.47 | 180.07 | 1 |
| tyrosine | 0.055 | 1.17 | 1.90 | 182.08 | 2 |
| bis(2-ethylhexyl)phthalate | 0.069 | 1.48 | 9.85 | 391.28 | 2 |
| 4-coumarate | 0.085 | 1.16 | 1.94 | 165.05 | 2 |
| pipecolate | 0.096 | 0.50 | 0.98 | 130.09 | 2 |
| valine | 0.103 | 0.77 | 0.85 | 118.09 | 2 |
| homocysteine | 0.123 | 0.87 | 1.11 | 136.04 | 2 |
| citrate | 0.131 | 1.47 | 1.16 | 191.02 | 2 |
| suberate | 0.151 | 0.93 | 3.88 | 173.08 | 1 |
| 5-hydroxytryptophan | 0.161 | 1.28 | 2.13 | 221.09 | 2 |
| dihydroxyacetone phosphate | 0.163 | 1.05 | 0.67 | 171.01 | 2 |
| pantothenate | 0.176 | 1.07 | 2.51 | 220.12 | 2 |
| phosphorylcholine | 0.205 | 0.71 | 0.82 | 184.07 | 1 |
| 4-guanidinobutanoate | 0.205 | 0.52 | 1.11 | 146.09 | 2 |
| diethanolamine | 0.207 | 1.52 | 0.84 | 106.09 | 1 |
| beta-alanine | 0.213 | 1.61 | 0.92 | 90.05 | 2 |
| 4-hydroxybenzoate | 0.217 | 0.64 | 3.30 | 137.02 | 2 |
| succinate semialdehyde | 0.233 | 1.05 | 0.83 | 103.04 | 1 |
| spermine | 0.235 | 0.72 | 0.67 | 203.22 | 1 |
| galactarate | 0.243 | 1.06 | 1.12 | 209.03 | 1 |
| n-formyl-l-methionine | 0.244 | 0.61 | 2.91 | 176.04 | 2 |
| l-ornithine | 0.246 | 1.04 | 0.70 | 133.10 | 2 |
| histidine | 0.271 | 1.00 | 0.80 | 156.08 | 1 |
| glycochenodeoxycholate | 0.287 | 0.66 | 6.82 | 450.32 | 2 |
| leucine | 0.291 | 0.73 | 1.94 | 132.10 | 2 |
| n-acetylglutamate | 0.291 | 0.96 | 1.86 | 188.06 | 1 |
| glutamate | 0.298 | 1.28 | 0.84 | 148.06 | 1 |
| histidine | 0.300 | 1.19 | 0.80 | 154.06 | 1 |
| cystine | 0.323 | 1.26 | 0.81 | 241.03 | 1 |
| ethanolamine phosphate | 0.326 | 0.76 | 0.82 | 140.01 | 1 |
| creatine | 0.335 | 0.95 | 0.91 | 132.08 | 2 |
| aspartate | 0.339 | 1.45 | 0.96 | 134.04 | 1 |
| n,n-dimethyl-arginine | 0.340 | 0.78 | 0.93 | 203.15 | 1 |
| ureidopropionate | 0.346 | 0.80 | 1.22 | 133.06 | 2 |
| 2-aminoisobutyrate | 0.347 | 1.22 | 0.83 | 104.07 | 2 |
| 3-methoxytyrosine | 0.347 | 0.94 | 2.15 | 212.09 | 1 |
| n-acetylputrescine | 0.349 | 0.83 | 1.09 | 131.12 | 2 |
| urate | 0.355 | 1.23 | 1.51 | 169.04 | 2 |
| indole-3-acetate | 0.357 | 1.13 | 3.64 | 176.07 | 1 |
| spermidine | 0.360 | 0.58 | 0.65 | 146.17 | 1 |
| 10-hydroxydecanoate | 0.366 | 1.30 | 6.69 | 187.13 | 1 |
| indole | 0.374 | 0.80 | 2.86 | 116.05 | 2 |
| glycocholate | 0.380 | 0.63 | 5.79 | 466.32 | 2 |
| theophylline | 0.386 | 0.21 | 2.76 | 181.07 | 1 |
| aminoisobutanoate | 0.393 | 1.08 | 0.89 | 104.07 | 1 |
| 1-aminocyclopropanecarboxylate | 0.398 | 1.30 | 0.83 | 102.05 | 1 |
| allantoin | 0.399 | 0.65 | 0.91 | 157.04 | 2 |
| 2-hydroxy-4-(methylthio)butanoate | 0.420 | 1.05 | 2.95 | 149.03 | 2 |
| glyceraldehyde | 0.425 | 0.94 | 1.43 | 89.02 | 2 |
| d-alanine | 0.426 | 1.36 | 0.80 | 88.04 | 2 |
| n-acetylaspartate | 0.435 | 0.63 | 1.49 | 174.04 | 1 |
| phenylalanine | 0.440 | 1.22 | 2.41 | 166.09 | 2 |
| cortisol | 0.473 | 1.06 | 5.13 | 363.22 | 2 |
| kynurenine | 0.476 | 1.04 | 2.41 | 209.09 | 2 |
| inosine monophosphate | 0.483 | 0.33 | 1.53 | 347.04 | 1 |
| succinate | 0.485 | 1.32 | 1.93 | 117.02 | 1 |
| trigonelline | 0.493 | 0.62 | 0.92 | 138.05 | 1 |
| n-acetylglucosamine | 0.500 | 0.42 | 0.94 | 222.10 | 1 |
| uridine | 0.505 | 0.78 | 1.87 | 245.08 | 1 |
| betaine | 0.512 | 1.00 | 1.18 | 118.09 | 2 |
| n-methylglutamate | 0.567 | 0.79 | 0.95 | 162.08 | 1 |
| tryptophan | 0.573 | 0.99 | 2.81 | 205.10 | 2 |
| deoxycholate | 0.581 | 0.45 | 7.77 | 391.29 | 2 |
| l-alanine | 0.585 | 1.28 | 0.81 | 90.05 | 2 |
| palmitoylcarnitine | 0.599 | 0.87 | 7.85 | 400.34 | 2 |
| citrulline | 0.610 | 1.04 | 0.85 | 176.10 | 1 |
| l-carnitine | 0.624 | 0.82 | 0.85 | 162.11 | 1 |
| alpha-hydroxyisobutyrate | 0.625 | 0.80 | 1.98 | 103.04 | 1 |
| threonine | 0.632 | 1.28 | 0.83 | 120.07 | 1 |
| hypoxanthine | 0.639 | 0.76 | 1.54 | 137.05 | 1 |
| xanthine | 0.647 | 0.78 | 1.72 | 153.04 | 1 |
| n-acetylneuraminate | 0.658 | 0.88 | 1.01 | 310.11 | 1 |
| isocitrate | 0.668 | 1.20 | 0.72 | 193.03 | 2 |
| glucuronate | 0.681 | 0.91 | 0.84 | 193.04 | 1 |
| glutarylcarnitine | 0.689 | 1.00 | 2.08 | 276.14 | 2 |
| indole-3-methyl acetate | 0.690 | 0.39 | 5.23 | 190.09 | 2 |
| methyl 4-aminobutyrate | 0.691 | 0.72 | 1.21 | 116.07 | 1 |
| glycerate | 0.707 | 0.63 | 0.96 | 105.02 | 2 |
| oxoproline | 0.731 | 1.04 | 0.83 | 130.05 | 1 |
| glucosamine 6-phosphate | 0.736 | 0.84 | 0.95 | 260.05 | 1 |
| creatinine | 0.739 | 0.85 | 0.87 | 114.07 | 1 |
| galactose 1-phosphate | 0.803 | 0.92 | 0.87 | 261.04 | 1 |
| glutamine | 0.809 | 1.07 | 0.81 | 147.08 | 1 |
| n-acetylleucine | 0.817 | 0.90 | 2.80 | 174.11 | 2 |
| nicotinamide | 0.821 | 1.09 | 1.52 | 123.06 | 1 |
| trans-4-hydroxy-l-proline | 0.826 | 1.06 | 0.83 | 132.07 | 2 |
| taurine | 0.832 | 0.83 | 0.82 | 126.02 | 1 |
| deoxyadenosine | 0.836 | 0.74 | 0.89 | 252.11 | 2 |
| n-acetylalanine | 0.857 | 0.84 | 1.98 | 132.07 | 1 |
| proline | 0.883 | 1.03 | 0.93 | 116.07 | 1 |
| sphinganine | 0.885 | 0.65 | 7.06 | 302.31 | 1 |
| lactate | 0.893 | 0.59 | 1.23 | 89.02 | 2 |
| lauroylcarnitine | 0.899 | 0.70 | 6.68 | 344.28 | 1 |
| hypotaurine | 0.907 | 1.01 | 0.81 | 110.03 | 1 |
| quinate | 0.908 | 0.34 | 0.97 | 191.06 | 1 |
| isoleucine | 0.909 | 0.61 | 1.77 | 132.10 | 2 |
| gluconate | 0.923 | 1.15 | 0.85 | 195.05 | 1 |
| methionine | 0.930 | 0.33 | 1.24 | 148.04 | 1 |
| serine | 0.934 | 1.48 | 0.80 | 106.05 | 2 |
| malate | 0.934 | 1.36 | 1.15 | 133.01 | 2 |
| n-acetylproline | 0.935 | 0.84 | 1.95 | 158.08 | 2 |
| benzoate | 0.963 | 1.34 | 3.82 | 121.03 | 2 |
| deoxycarnitine | 0.981 | 0.78 | 0.96 | 146.12 | 2 |
| n-alpha-acetyllysine | 0.982 | 1.07 | 1.22 | 189.12 | 2 |
| pyroglutamate | 0.983 | 0.73 | 1.62 | 130.05 | 2 |
| 1-methyladenosine | 0.988 | 1.12 | 1.24 | 282.12 | 1 |
| asparagine | 0.993 | 1.43 | 0.81 | 133.06 | 2 |

*P*-value = The *P*-value of the fixed effect term in linear mixed-effects model. The differential metabolites were marked with bold.

**Supplemental Table 3.** ROC analyses of random forest model to predict the immune response to ART

| **Model** | **n** | **AUC** | **Sensitivity** | **Specificity** |
| --- | --- | --- | --- | --- |
| **Pre-ART** |  |  |  |  |
| CD4 | 115 | 0.74 (0.63, 0.83) | 0.79 (0.48, 0.92) | 0.69 (0.55, 0.94) |
| Metabolites | 115 | 0.66 (0.55, 0.76) | 0.62 (0.44, 0.85) | 0.76 (0.46, 0.90) |
| CD4 & Metabolites | 115 | 0.84 (0.75, 0.91) | 0.71 (0.52, 0.88) | 0.91 (0.76, 1.00) |
| **Dynamic metabolic biomarkers** |  |  |  |  |
| Metabolites (pre-ART & Δ_12-0w_) & CD4 (pre-ART & 12-week) | 115 | 0.86 (0.78, 0.94) | 0.81 (0.67, 0.94) | 0.90 (0.73, 0.99) |
| Metabolites (pre-ART & Δ_24-0w_) & CD4 (pre-ART & 24-week) | 115 | 0.88 (0.80, 0.95) | 0.79 (0.62, 0.94) | 0.93 (0.75, 0.99) |
| Metabolites (pre-ART & Δ_36-0w_) & CD4 (pre-ART & 36-week) | 115 | 0.90 (0.83, 0.95) | 0.83 (0.69, 0.94) | 0.90 (0.79, 0.97) |
| Metabolites (pre-ART & Δ_48-0w_) & CD4 (pre-ART & 48-week) | 114 | 0.87 (0.78, 0.94) | 0.74 (0.62, 0.89) | 0.97 (0.81, 1.00) |
| Metabolites (pre-ART & Δ_72-0w_) & CD4 (pre-ART & 72-week) | 107 | 0.87 (0.79, 0.94) | 0.85 (0.72, 0.96) | 0.89 (0.75, 0.97) |

ROC = receiver operating characteristic, AUC = area under curve.

* The combination of metabolites includes lumichrome, 1-methyl-l-histidine, arginine, n,n,n-trimethyllysine, lysine.

**Supplemental Table 4.** Result from pathway analysis

| **Pathway** | **Total** | **Expected** | **Hits** | ***P*** | **FDR** | **Impact** |
| --- | --- | --- | --- | --- | --- | --- |
| **alanine, aspartate and glutamate metabolism** | **28** | **1.30** | **10** | **<0.001** | **<0.001** | **0.67** |
| **arginine biosynthesis** | **14** | **0.65** | **7** | **<0.001** | **<0.001** | **0.51** |
| **arginine and proline metabolism** | **36** | **1.67** | **10** | **<0.001** | **<0.001** | **0.46** |
| **beta-Alanine metabolism** | **21** | **0.98** | **6** | **<0.001** | **0.004** | **0.56** |
| **valine, leucine and isoleucine biosynthesis** | **8** | **0.37** | **4** | **<0.001** | **0.004** | **0.00** |
| **glyoxylate and dicarboxylate metabolism** | **32** | **1.49** | **7** | **<0.001** | **0.006** | **0.17** |
| **pantothenate and CoA biosynthesis** | **20** | **0.93** | **5** | **0.002** | **0.019** | **0.10** |
| histidine metabolism | 16 | 0.74 | 4 | 0.005 | 0.051 | 0.22 |
| glutathione metabolism | 28 | 1.30 | 5 | 0.008 | 0.072 | 0.03 |
| citrate cycle (TCA cycle) | 20 | 0.93 | 4 | 0.012 | 0.088 | 0.21 |
| phenylalanine, tyrosine and tryptophan biosynthesis | 4 | 0.19 | 2 | 0.012 | 0.088 | 1.00 |
| glycine, serine and threonine metabolism | 33 | 1.53 | 5 | 0.016 | 0.108 | 0.29 |
| nitrogen metabolism | 6 | 0.28 | 2 | 0.028 | 0.158 | 0.00 |
| butanoate metabolism | 15 | 0.70 | 3 | 0.029 | 0.158 | 0.03 |
| one carbon pool by folate | 26 | 1.21 | 4 | 0.030 | 0.158 | 0.26 |
| purine metabolism | 70 | 3.25 | 7 | 0.040 | 0.200 | 0.17 |
| lysine degradation | 30 | 1.39 | 4 | 0.047 | 0.210 | 0.00 |
| phenylalanine metabolism | 8 | 0.37 | 2 | 0.050 | 0.210 | 0.36 |
| taurine and hypotaurine metabolism | 8 | 0.37 | 2 | 0.050 | 0.210 | 0.83 |
| pyrimidine metabolism | 39 | 1.81 | 4 | 0.104 | 0.415 | 0.05 |
| tryptophan metabolism | 41 | 1.91 | 4 | 0.119 | 0.454 | 0.37 |
| amino sugar and nucleotide sugar metabolism | 42 | 1.95 | 4 | 0.127 | 0.463 | 0.21 |
| nicotinate and nicotinamide metabolism | 15 | 0.70 | 2 | 0.152 | 0.527 | 0.19 |
| glycerolipid metabolism | 16 | 0.74 | 2 | 0.168 | 0.561 | 0.09 |
| sphingolipid metabolism | 32 | 1.49 | 3 | 0.183 | 0.587 | 0.08 |
| cysteine and methionine metabolism | 33 | 1.53 | 3 | 0.195 | 0.601 | 0.26 |
| ubiquinone and other terpenoid-quinone biosynthesis | 19 | 0.88 | 2 | 0.220 | 0.652 | 0.00 |
| glycerophospholipid metabolism | 36 | 1.67 | 3 | 0.232 | 0.664 | 0.05 |
| propanoate metabolism | 22 | 1.02 | 2 | 0.273 | 0.726 | 0.00 |
| valine, leucine and isoleucine degradation | 40 | 1.86 | 3 | 0.284 | 0.726 | 0.00 |
| pyruvate metabolism | 23 | 1.07 | 2 | 0.290 | 0.726 | 0.03 |
| pentose phosphate pathway | 23 | 1.07 | 2 | 0.290 | 0.726 | 0.04 |
| glycolysis or Gluconeogenesis | 26 | 1.21 | 2 | 0.343 | 0.798 | 0.00 |
| vitamin B6 metabolism | 9 | 0.42 | 1 | 0.349 | 0.798 | 0.00 |
| ascorbate and aldarate metabolism | 9 | 0.42 | 1 | 0.349 | 0.798 | 0.52 |
| primary bile acid biosynthesis | 46 | 2.14 | 3 | 0.362 | 0.804 | 0.03 |
| biotin metabolism | 10 | 0.46 | 1 | 0.380 | 0.821 | 0.00 |
| inositol phosphate metabolism | 30 | 1.39 | 2 | 0.411 | 0.865 | 0.00 |
| d-Amino acid metabolism | 15 | 0.70 | 1 | 0.512 | 1.000 | 0.00 |
| pentose and glucuronate interconversions | 19 | 0.88 | 1 | 0.597 | 1.000 | 0.10 |
| selenocompound metabolism | 20 | 0.93 | 1 | 0.616 | 1.000 | 0.00 |
| fructose and mannose metabolism | 20 | 0.93 | 1 | 0.616 | 1.000 | 0.00 |
| galactose metabolism | 27 | 1.26 | 1 | 0.726 | 1.000 | 0.03 |
| porphyrin metabolism | 31 | 1.44 | 1 | 0.775 | 1.000 | 0.00 |
| glycosylphosphatidylinositol (GPI)-anchor biosynthesis | 32 | 1.49 | 1 | 0.785 | 1.000 | 0.00 |
| fatty acid degradation | 39 | 1.81 | 1 | 0.847 | 1.000 | 0.00 |
| tyrosine metabolism | 42 | 1.95 | 1 | 0.868 | 1.000 | 0.14 |
| steroid hormone biosynthesis | 87 | 4.04 | 1 | 0.986 | 1.000 | 0.03 |

Hits = the matched number of metabolites in the pathway;

Impact = the pathway impact value calculated from pathway topology analysis.


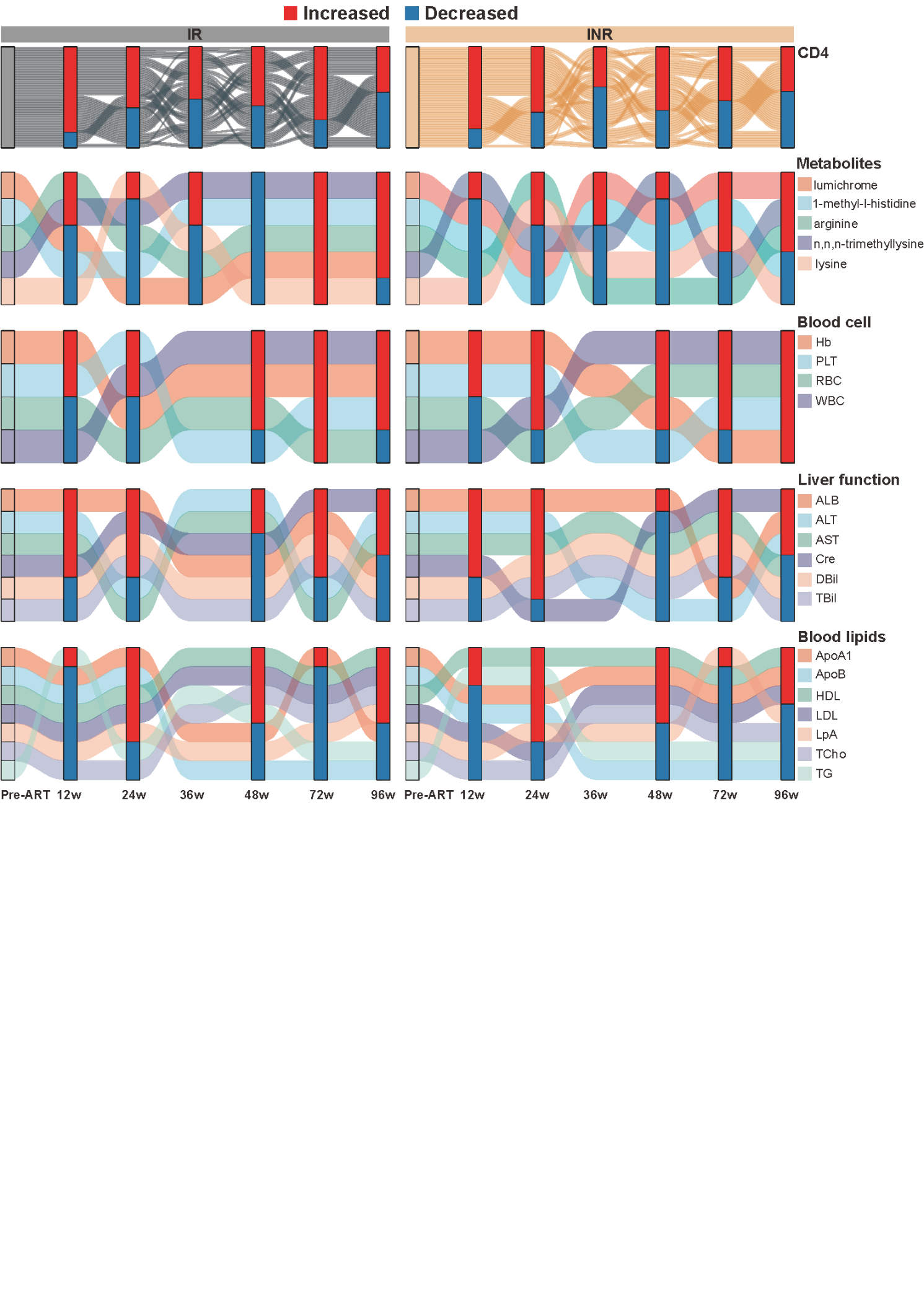


**Supplemental Figure 1.** Different temporal patterns of dynamic differential metabolites and clinical characteristics between IR and INR.

The first row shows the CD4 count trends for 116 PLHIVs in the IR group (left) and the INR group (right). The increases (red) and decreases (blue) in this row are determined based on whether the CD4 count at the current time point has increased or decreased compared to the previous time point. The increases and decreases of metabolites and other clinical characteristics (except for CD4) are determined based on the average difference between the current time point and the previous time point. The specific order of metabolites and clinical characteristics within the increased or decreased categories is arranged randomly for visualization purposes and does not reflect the magnitude of change.
